# Clinical Validation of Tissue and Liquid Companion Diagnostics for *BRAF* V600E Detection in Non–Small Cell Lung Cancers from the PHAROS Study

**DOI:** 10.1158/2767-9764.CRC-26-0102

**Published:** 2026-07-29

**Authors:** Gregory J. Riely, Jean-François Martini, Norberto Pantoja Galicia, Brian Tunquist, Keith Wilner, Richard S.P. Huang, Cui Guo, Shibing Deng, Wei Meng, Jimmy Kiely, Linda Zhou, David L. Smith, Mark Hartman, Chenming Cui, Nada Rifi, Bruce E. Johnson, Anne S. Tsao

**Affiliations:** 1 Memorial Sloan Kettering Cancer Center, New York, New York.; 2 Pfizer, La Jolla, California.; 3 Foundation Medicine, Inc., Boston, Massachusetts.; 4 Pfizer, Boulder, Colorado.; 5 Pfizer, Paris, France.; 6 Dana-Farber Cancer Institute, Boston, Massachusetts.; 7 The University of Texas MD Anderson Cancer Center, Houston, Texas.

## Abstract

The detection of biomarkers in blood or tumor tissue samples may affect the efficacy of targeted therapy in patients with non–small cell lung cancer (NSCLC). The FoundationOne Liquid CDx (F1LCDx) and tissue FoundationOne CDx (F1CDx) companion diagnostic tests were approved by the US Food and Drug Administration to identify patients with *BRAF* V600E–mutant metastatic NSCLC who may benefit from encorafenib combined with binimetinib. This clinical bridging study aimed to establish F1LCDx and F1CDx as companion diagnostics for the identification of patients with *BRAF* V600E–mutant metastatic NSCLC and to determine concordance between these tests and clinical trial assays (CTA) in samples from patients enrolled in the phase II PHAROS trial. In samples with sufficient circulating tumor DNA (ctDNA; defined as tumor fraction ≥1%), F1LCDx testing revealed a positive percent agreement (PPA) of 83.3% (Wilson score two-sided 95% confidence interval, 68.1%–92.1%). F1CDx testing revealed a PPA of 93.2% (85%–97%), suggesting that F1CDx may robustly identify patients with *BRAF* V600E–mutant NSCLC and complement F1LCDx testing in this population. The efficacy of encorafenib plus binimetinib in patients selected by F1LCDx or F1CDx was based on the objective response rate (ORR) by independent radiology review in the PHAROS trial. The estimated ORRs in the F1LCDx+/CTA+ population were 60.4% versus 63.3% in the CTA+ population. Similarly, the estimated ORRs in the F1CDx+/CTA+ population were 70.6% versus 64.1% in the CTA+ population. These findings suggest that both tests may be clinically useful in predicting the efficacy of encorafenib plus binimetinib in patients with metastatic NSCLC.

**Significance:**

This clinical bridging study assessed F1LCDx and tissue F1CDx companion diagnostic tests for identifying patients with *BRAF* V600E–mutant metastatic NSCLC. Results support both tests in identifying patients and predicting an objective response in patients who may be eligible for treatment with encorafenib combined with binimetinib.

## Introduction

The detection of biomarkers in blood or tumor tissue samples from patients with advanced-stage or metastatic non–small cell lung cancer (NSCLC) can help predict a beneficial therapeutic response. The detection of specific biomarkers may dictate treatment decision-making, including the use of chemotherapy, immunotherapy, and/or targeted therapy (1). Although a tissue biopsy remains the gold standard for lung cancer diagnosis, scarcity of tumor tissue is a commonly encountered challenge and may require patients to undergo more than one tissue biopsy in order to achieve a definitive diagnosis (e.g., approximately 50%–60% of patients with advanced NSCLC may require a re-biopsy; refs. 1–4). In some cases, a tissue biopsy may not be feasible, biopsy procedures may be expensive, or additional time may be required to complete scheduling and comprehensive profiling analysis prior to treatment initiation. Liquid biopsy offers several advantages over tissue biopsy; it is repeatable, minimally invasive, and cost-effective and can be analyzed within a shorter turnaround time (4–6). An archived tissue biopsy reflects static tumor information; however, a liquid biopsy allows for multiple serial sample collections, which can be useful for reflecting real-time tumor information, detecting disease progression, monitoring treatment response, and identifying mutations or potential biomarkers of resistance or sensitivity (7, 8). Moreover, comprehensive genomic profiling of circulating tumor DNA (ctDNA) in clinical trials has resulted in significantly reduced screening time and improved trial enrollment without compromising treatment efficacy compared with tissue genotyping (9). Recent NSCLC guidelines recommend the use of plasma-based liquid biopsy approaches, including plasma ctDNA assays, in conjunction with tissue-based testing for genotyping select biomarkers (10, 11).

Several ctDNA-based companion diagnostic assays have been approved by the US Food and Drug Administration (FDA) to detect oncogenic driver mutations in patients with NSCLC (12). The FDA approval of the FoundationOne CDx (F1CDx) and FoundationOne Liquid CDx (F1LCDx) tests represents the first simultaneous approval of companion diagnostics to identify patients with B-Raf proto-oncogene, serine/threonine kinase (*BRAF*) V600E–mutated metastatic NSCLC who may benefit from encorafenib in combination with binimetinib (13).

Oncogenic driver mutations such as alterations in the *BRAF* gene are found in approximately 3% to 5% of NSCLC cases, with *BRAF* V600E mutations present in nearly half of these cases (14–16). Patients with *BRAF*-mutant metastatic NSCLC derive limited benefits from treatment with platinum-based chemotherapy or immunotherapy compared with patients without *BRAF* alterations (15). Although BRAF inhibitor monotherapy was efficacious in patients with *BRAF* V600E–mutant NSCLC, its clinical activity was limited by acquired resistance and reactivation of the mitogen-activated protein kinase (MAPK) pathway (17). Dual inhibition of the MAPK pathway with the combination of BRAF and MAPK kinase (MEK) inhibitors in these patients extended antitumor activity and had a tolerable safety profile (14, 18). On the basis of recent guidelines, targeted therapy containing both a BRAF inhibitor and a MEK inhibitor (e.g., encorafenib plus binimetinib) is the preferred first-line treatment for patients with *BRAF* V600E–mutant metastatic NSCLC (19).

The PHAROS trial (NCT03915951) is an ongoing, single-arm, open-label, multicenter, phase II trial evaluating the efficacy and safety of the combination of encorafenib, a *BRAF* V600–mutant kinase inhibitor, and binimetinib, a MEK1/2 inhibitor, in two cohorts: treatment-naïve patients and those with previously treated *BRAF* V600–mutated metastatic NSCLC (14). In the trial, patients received oral encorafenib 450 mg once daily plus oral binimetinib 45 mg twice daily in 28-day cycles (14). The combination showed clinically meaningful activity in both treatment-naïve and previously treated patients {objective response rate (ORR) by independent radiology review (IRR): 75% [95% confidence interval (CI), 62%–85%] and 46% (30%–63%), respectively; median progression-free survival: 30.2 months (15.7–not estimable) and 9.3 months (6.2–24.8), respectively; and median overall survival: 47.6 months (95% CI, 31.3–not estimable) and 22.7 months (95% CI, 14.1–32.6), respectively; refs. 20, 21}. The PHAROS trial used clinical trial assays (CTAs) to identify and enroll patients with *BRAF* V600 class I mutations in tumor tissue or blood samples as determined by polymerase chain reaction (PCR)– or next-generation sequencing (NGS)–based local testing (14). Archived tumor tissue or a newly obtained core or excisional biopsy sample from all patients was required to be submitted to the central testing laboratory for confirmatory testing.

Through a retrospective analysis of banked samples from patients with metastatic NSCLC enrolled in the PHAROS trial, this clinical bridging study aimed to establish F1LCDx and F1CDx as companion diagnostics for the detection of the *BRAF* V600E alteration and to determine the concordance between these tests and CTAs in this patient population.

## Materials and Methods

### Clinical bridging study design and samples

This study evaluated the clinical validity of F1LCDx (Foundation Medicine, Inc.; RRID: SCR_025628) and F1CDx (Foundation Medicine, Inc.; RRID: SCR_025628) as companion diagnostic tests for identifying patients with *BRAF* V600E–mutant metastatic NSCLC using plasma and tumor tissue samples, respectively, from the PHAROS trial. Patients in the clinical trial were stratified by prior treatment into treatment-naïve and previously treated cohorts. Only patients with available plasma and tumor tissue samples were analyzed in this clinical bridging study. The presence of a *BRAF* V600E mutation in tumor tissue or blood was established by PCR- or NGS-based local assay results (i.e., the CTA). Tumor tissue for confirmatory *BRAF* V600 mutation status could not be obtained from previously irradiated sites and had to consist of 1 block or a minimum of 8 unstained slides of analyzable tissue. F1LCDx testing was performed on plasma samples from patients in the PHAROS trial who tested positive for *BRAF* V600E in tumor tissue or blood by the CTA (i.e., CTA+). Additional commercial *BRAF* V600E–negative tissue samples from patients with metastatic NSCLC and matched plasma (CTA−) were tested using the University of Washington (UW) OncoPlex NGS assay (UW School of Medicine; RRID: SCR_006147) or cobas PCR assay (Roche Diagnostics; RRID: SCR_025096). The remaining *BRAF* V600E–negative samples were plasma samples previously tested by the TruSight Oncology 500 ctDNA assay. This study involved retrospective testing of plasma samples from the PHAROS trial; as such, no additional patient follow-up was conducted.

Assessment of detection with F1LCDx was performed without knowledge of the CTA results. The analysis plan was developed without prior knowledge of clinical information. Assessment of concordance was performed as prespecified and independently of the clinical information. Clinical information was available for the assessment of clinical validity.

The F1LCDx is a pan-cancer, *in vitro* diagnostic test intended to provide tumor mutation profiling in patients with solid malignant neoplasms that uses targeted high-throughput, hybridization-based capture technology to detect and report substitutions, insertions, and deletions in 311 genes in the plasma of anticoagulated peripheral whole blood (22). Samples of frozen plasma that met the minimum criteria for F1LCDx operational testing, had ≥20 ng of cell-free DNA input [as assessed by the TapeStation assay (Agilent; RRID: SCR_018435)] for primary analysis, and were obtained with appropriate consent and institutional review board oversight to allow clinical bridging were included in this study analysis.

**Figure 1.**
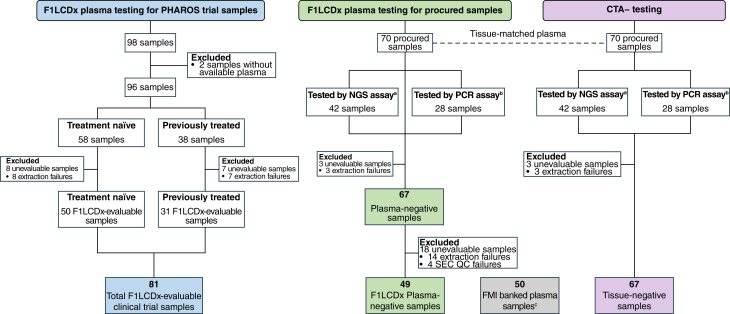
Plasma sample processing flow chart. FMI, Foundation Medicine, Inc.; QC, quality control; SEQ, sequencing; TSO500, TruSight Oncology 500 ctDNA Assay. ^a^UW OncoPlex NGS assay. ^b^cobas *BRAF* V600E PCR assay. ^c^Banked plasma samples were selected based on a TSO500-negative result for *BRAF* V600E and were previously tested with F1LCDx.

A total of 218 samples, including 98 plasma samples from patients participating in PHAROS and 120 commercial *BRAF* V600E–negative (CTA−) matched tissue and plasma samples, were included in the F1LCDx study (Fig. 1). The 96 clinical trial samples with available plasma that were evaluable by F1LCDx were from 58 treatment-naïve and 38 previously treated patients. The 120 commercial *BRAF* V600E–negative plasma samples consisted of 70 procured commercial tissue samples with matched plasma (42 tissue samples for UW OncoPlex testing and 28 tissue samples for cobas PCR assay testing) and 50 negative plasma samples previously tested by the TruSight Oncology 500 ctDNA assay (Illumina; RRID: SCR_010233). Three procured samples were not included in this clinical bridging analysis because they did not have enough material for external testing and were unevaluable for the CTA.

For the purposes of this analysis, the study populations were defined as follows:

1. CTA+: patients who were *BRAF* V600E positive as determined by the CTA.
  - F1LCDx+/CTA+ or F1CDx+/CTA+: patients who were *BRAF* V600E positive as determined by the CTA and the F1LCDx or F1CDx test.
  - F1LCDx−/CTA+ or F1CDx−/CTA+: patients who were *BRAF* V600E positive as determined by the CTA but *BRAF* V600E negative as determined by the F1LCDx or F1CDx test.
  - F1LCDx unevaluable/CTA+: patients who were *BRAF* V600E positive as determined by the CTA but unevaluable by the F1LCDx (e.g., includes samples that did not have plasma available for F1LCDx or failed F1LCDx testing).
2. CTA−: patients who were *BRAF* V600E negative as determined by the CTA.
  - F1LCDx−/CTA− or F1CDx−/CTA−: patients who were *BRAF* V600E negative as determined by the CTA and the F1LCDx or F1CDx test.
  - F1LCDx+/CTA− or F1CDx+/CTA−: patients who were *BRAF* V600E negative as determined by the CTA but *BRAF* V600E positive as determined by the F1LCDx or F1CDx test.
  - F1LCDx unevaluable/CTA−: patients who were *BRAF* V600E negative as determined by the CTA but unevaluable by the F1LCDx (e.g., includes samples that did not have plasma available for F1LCDx or failed F1LCDx testing).

Details on the clinical bridging study design and samples for F1CDx analyses are described in the Supplementary Methods.

### Concordance analysis

The concordance between the CTAs and the F1LCDx test was evaluated by the positive percent agreement (PPA), negative percent agreement (NPA), and Wilson score two-sided 95% CIs. R programming language was used for all statistical analyses (RRID: SCR_001905).

PPA for F1LCDx+/CTA+ patients was calculated using the formula:

$$PPA=100\times \frac{n11}{\left(n11+n10\right)}\%$$

where *n*11 is defined as the number of F1LCDx+/CTA+ samples and *n*10 is defined as the number of F1LCDx−/CTA+ samples (23).

NPA for F1LCDx−/CTA− patients was calculated using the formula:

$$NPA=100\times \frac{n00}{\left(n00+n01\right)}\%$$

where *n*00 is defined as the number of F1LCDx−/CTA− samples and *n*01 is defined as the number of F1LCDx+/CTA− samples (23). The prevalence-adjusted positive predictive values (PPV), negative predictive values (NPV), and the bootstrapping two-sided 95% CIs were calculated by adjusting for the prevalence of *BRAF* V600E mutations among the intention-to-treat population (24), with 2% (25), 4% (26), and 8% (27) as the estimated prevalences, respectively. The same concordance analysis, as detailed in this section, was conducted between the CTAs and the F1CDx test.

### Clinical validation

A description of the PHAROS study endpoints was published previously (14), and details are described in the Supplementary Methods. In the present analysis, the clinical validity of the F1LCDx and F1CDx tests was evaluated by assessing clinical efficacy in the F1LCDx and F1CDx *BRAF* V600E–mutant populations (F1LCDx+ and F1CDx+, respectively) based on the ORR by IRR for the combined and individual PHAROS cohorts according to treatment (i.e., treatment naïve and previously treated).

### Sensitivity analyses

To assess the robustness of the results subject to missing F1LCDx and F1CDx test results, sensitivity analyses were performed. The multiple imputation method plus bootstrapping (28, 29) was used to impute the F1LCDx and F1CDx *BRAF* V600E status for the F1LCDx-unevaluable and F1CDx-unevaluable patients in the enrolled populations (CTA+), respectively. The concordance analyses and clinical efficacies in F1LCDx+ and F1CDx+ patients were updated by accounting for the imputed data. Multiple imputation was conducted in the original dataset, and a total of 200 imputation datasets were generated. The PPA and PPV estimates were computed for each of the 200 imputed complete datasets.

### Statistical analyses

For the concordance analysis results, two-sided 95% CIs were calculated using the Wilson score method for PPA, NPA, and adjusted PPV. The bootstrapping method was used for the adjusted NPV. For the efficacy results, the ORR by IRR [i.e., patients who achieved a partial response (PR) or complete response (CR)] was calculated for each cohort separately and combined. The corresponding two-sided 95% CI was calculated based on the variance of the F1LCDx+ and F1CDx+ efficacy estimates.

### Ethical statement

The PHAROS trial was approved by the institutional review board or independent ethics committee at each center and was conducted in accordance with the requirements of the regulatory authorities of each country and with the provisions of the Declaration of Helsinki and the Good Clinical Practice guidelines of the International Council on Harmonization (14). All patients provided written informed consent.

## Results

Of 98 patients from the PHAROS trial, 96 had plasma samples available for F1LCDx plasma testing (Fig. 1). This pool of plasma samples comprised 58 samples from treatment-naïve patients and 38 from patients who received prior treatment. Eight plasma samples from the treatment-naïve and 7 from the previously treated groups were not evaluable. Thus, a total of 81 evaluable plasma samples from 98 clinical trial patients (CTA+) were available for F1LCDx testing.

**Figure 2.**
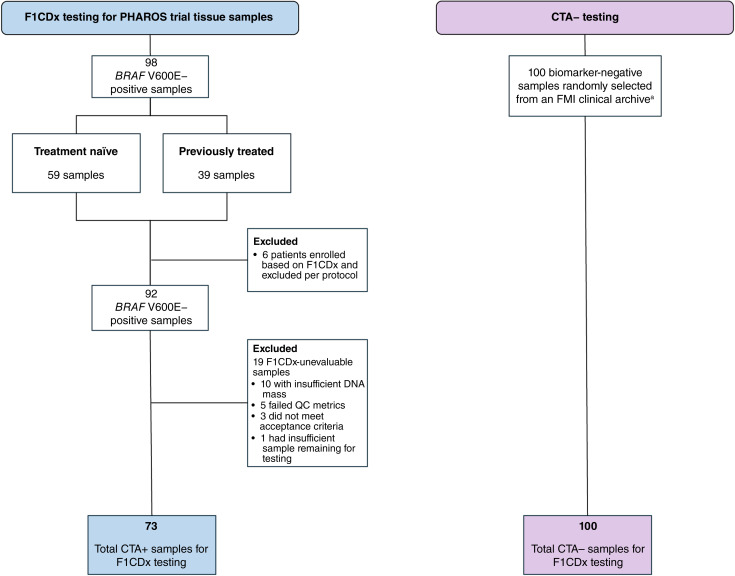
Tumor tissue sample processing flow chart. FMI, Foundation Medicine, Inc.; QC, quality control. ^a^Re-analyzed by the UW OncoPlex for the *BRAF* V600 biomarker.

All 98 patients from the PHAROS trial had *BRAF* V600E–positive (CTA+) tissue samples, comprising 59 samples from treatment-naïve patients and 39 from patients who received prior treatment (Fig. 2). Six patients were enrolled in the study based on the F1CDx assay result and were therefore excluded from the concordance analysis (CTAs vs. F1CDx) per the study protocol. Of the remaining 92 patients, 19 were excluded based on samples that were F1CDx unevaluable, including 3 patients who had tumor tissue samples that did not meet the minimum incoming sample acceptance criteria and 1 patient who did not have sufficient material remaining for F1CDx testing and was therefore included in the F1CDx-unevaluable population. Thus, 73 total *BRAF* V600E–positive (CTA+) tissue samples were evaluable for F1CDx testing.

### Baseline characteristics

The baseline demographics and clinical characteristics were similar between the populations from the F1LCDx-evaluable/CTA+ and F1LCDx-unevaluable/CTA+ groups for both treatment-naïve and previously treated patients (Table 1) and were reflective of the intention-to-treat population in PHAROS (14). Treatment-naïve patients had a median age of 68 and 70 years in the F1LCDx-evaluable/CTA+ (*n* = 50) and F1LCDx-unevaluable/CTA+ (*n* = 9) populations, respectively. The majority of patients had an Eastern Cooperative Oncology Group (ECOG) performance status of 1 in both populations (F1LCDx evaluable/CTA+, 68%; F1LCDx unevaluable/CTA+, 66.7%). Previously treated patients had a median age of 71 and 72 years in the F1LCDx-evaluable/CTA+ (*n* = 31) and F1LCDx-unevaluable/CTA+ (*n* = 8) populations, respectively. Most of the previously treated patients had an ECOG performance status of 1 in both populations (F1LCDx evaluable/CTA+, 83.9%; F1LCDx unevaluable/CTA+, 75%). Baseline demographics and clinical characteristics of the F1CDx populations are shown in Supplementary Table S1.

**Table 1.** Demographics and clinical characteristics in the F1LCDx-evaluable and -unevaluable populations.

Covariate	Treatment naïve		Previously treated	
	F1LCDx evaluable/CTA+ (*n* = 50)	F1LCDx unevaluable/CTA+ (*n* = 9)	F1LCDx evaluable/CTA+ (*n* = 31)	F1LCDx unevaluable/CTA+ (*n* = 8)
Age	​	​	​	​
Median (range), years	68 (51–83)	70 (47–82)	71 (53–86)	72 (55–83)
Sex, *n* (%)	​	​	​	​
Female	29 (58)	4 (44.4)	18 (58.1)	1 (12.5)
Male	21 (42)	5 (55.6)	13 (41.9)	7 (87.5)
Race, *n* (%)	​	​	​	​
American Indian/Alaskan Native	1 (2)	0	0	0
Asian	3 (6)	0	4 (12.9)	0
Black/African American	1 (2)	0	2 (6.5)	0
White	44 (88)	9 (100)	25 (80.7)	8 (100)
Unknown	1 (2)	0	0	0
ECOG PS, *n* (%)	​	​	​	​
0	16 (32)	3 (33.3)	5 (16.1)	2 (25)
1	34 (68)	6 (66.7)	26 (83.9)	6 (75)
Smoking status, *n* (%)	​	​	​	​
Current smoker	5 (10)	3 (33.3)	2 (6.5)	3 (37.5)
Former smoker	28 (56)	5 (55.6)	18 (58.1)	5 (62.5)
Never smoked	17 (34)	1 (11.1)	11 (35.5)	0
Tissue sample site, *n* (%)	​	​	​	​
Metastatic	18 (36)	2 (22.2)	12 (38.7)	2 (25)
Primary	19 (38)	5 (55.6)	14 (45.2)	2 (25)
Not available	13 (26)	2 (22.2)	5 (16.1)	4 (50)
Tissue handling, *n* (%)	​	​	​	​
Core needle/excisional biopsy	24 (48)	6 (66.7)	21 (67.7)	6 (75)
Fine needle aspiration	12 (24)	1 (11.1)	5 (16.1)	1 (12.5)
Resection	9 (18)	2 (22.2)	3 (9.7)	1 (12.5)
Other	3 (6)	0	2 (6.5)	0
Not available	2 (4)	0	0	0
TF status, *n* (%)	​	​	​	​
<1%	28 (56)	0	17 (54.8)	0
≥1%	22 (44)	0	14 (45.2)	0
Unevaluable	0	9 (100)	0	8 (100)

Abbreviation: ECOG PS, ECOG performance status.

### Concordance between the CTA and the F1LCDx test

To assess the concordance between the CTA and the F1LCDx test in detecting *BRAF* V600E in plasma samples, a total of 215 samples were included in the clinical bridging analysis, which consisted of 98 CTA+ plasma samples from patients enrolled in the PHAROS trial and 117 CTA− commercially procured plasma samples. Among these 215 samples, 213 had available plasma for F1LCDx testing, and 180 of the 213 (84.5%) samples yielded valid F1LCDx testing results (Supplementary Table S2).

Of a total of 98 CTA+ samples, 81 were evaluable for F1LCDx testing (50 samples from treatment naïve and 31 from previously treated patients; Fig. 1). Of the 81 F1LCDx-evaluable samples, 48 were positive (F1LCDx+) and 33 were negative (F1LCDx−) for *BRAF* V600E (Table 2). For a tumor fraction (TF) of <1% or ≥1%, the PPA was 40.9% (Wilson score two-sided 95% CI, 27.7%–55.6%) and 83.3% (Wilson score two-sided 95% CI, 68.1%–92.1%), respectively (Table 3). The resulting PPA for the whole population was 59.3% (Wilson score two-sided 95% CI, 48.4%–69.3%; Supplementary Table S3). Among 117 commercially procured plasma samples that were negative for *BRAF* V600E by the CTA (CTA−), 99 CTA− samples were F1LCDx evaluable and were included in the concordance analysis (Table 2). All 99 (100%) were also found to be *BRAF* V600E negative by the F1LCDx test (F1LCDx−/CTA−). Thus, the resulting NPA for the whole population was 100% (Wilson score two-sided 95% CI, 96.3%–100%; Supplementary Table S3).

**Table 2.** Comparison of *BRAF* V600E status between the CTA and F1LCDx.

​F1LCDx status	CTA+			CTA−			Total^a^		
	Overall	TF < 1%	TF ≥ 1%	Overall	TF < 1%	TF ≥ 1%	Overall	TF < 1%	TF ≥ 1%
F1LCDx evaluable	81	44	36	99	56	43	180	100	79
F1LCDx+	48	18	30	0	0	0	48	18	30
F1LCDx−	33	26	6	99	56	43	132	82	49
F1LCDx unevaluable	17	0	0	18	0	0	35	0	0
Total	98	44	36	117	56	43	215	100	79

a Only F1LCDx-evaluable samples are included as the F1LCDx-unevaluable samples do not have valid TF scores. Additionally, 1 F1LCDx−/CTA+ sample was excluded as its TF score could not be determined.

**Table 3.** Concordance analysis results between CTA and F1LCDx by TF and CTA and F1CDx.

​F1LCDx subgroup and concordance measure	Prevalence, %	Concordant result with CTA and F1LCDx test	Denominator^a^	Point estimate, % (two-sided 95% CI^b^)
F1LCDx, TF <1%				
PPA	NA	18	44	40.9 (27.7–55.6)
NPA	NA	56	56	100 (93.6–100)
Adjusted PPV	2	NA	NA	100 (100–100)
Adjusted NPV	2	NA	NA	98.8 (98.5–99.1)
Adjusted PPV	4	NA	NA	100 (100–100)
Adjusted NPV	4	NA	NA	97.6 (97.1–98.1)
Adjusted PPV	8	NA	NA	100 (100–100)
Adjusted NPV	8	NA	NA	95.1 (94.1–96.2)
F1LCDx, TF ≥1%				
PPA	NA	30	36	83.3 (68.1–92.1)
NPA	NA	43	43	100 (91.8–100)
Adjusted PPV	2	NA	NA	100 (100–100)
Adjusted NPV	2	NA	NA	99.7 (99.4–99.9)
Adjusted PPV	4	NA	NA	100 (100–100)
Adjusted NPV	4	NA	NA	99.3 (98.7–99.8)
Adjusted PPV	8	NA	NA	100 (100–100)
Adjusted NPV	8	NA	NA	98.6 (97.4–99.5)
F1CDx				
PPA	NA	68^c^	73	93.2 (85–97)
NPA	NA	100	100	100 (96.3–100)
Adjusted PPV	2	NA	NA	100 (94.7–100)
Adjusted NPV	2	NA	NA	99.9 (99.7–100)
Adjusted PPV	4	NA	NA	100 (94.7–100)
Adjusted NPV	4	NA	NA	99.7 (99.4–99.9)
Adjusted PPV	8	NA	NA	100 (94.7–100)
Adjusted NPV	8	NA	NA	99.4 (98.8–99.9)

Abbreviation: NA, not available.

a The denominator for PPA is the total number of CTA+ samples among the F1LCDx- or F1CDx-evaluable samples. The denominator for NPA is the total number of CTA− samples among the F1LCDx- or F1CDx-evaluable samples.

b CI was calculated using the Wilson score method for PPA, NPA, and adjusted PPV while using the bootstrap method for the adjusted NPV.

c Four of the 5 samples that were F1CDx−/CTA+ would have had a qualified F1CDx test report because of sample quality issues. A qualified report indicates that sensitivity for variant detection, including short variants, is potentially reduced. In the clinical setting, this will be stated on the clinical report and repeat testing is recommended.

### Concordance between the CTA and the F1CDx test

To assess the concordance between the CTA and F1CDx test in detecting *BRAF* V600E in tumor tissue, a total of 198 tissue samples were included in the clinical bridging analysis, which consisted of 98 CTA+ tissue samples from patients enrolled in the PHAROS trial and 100 CTA− tissue samples randomly selected from the clinical archive of Foundation Medicine, Inc. Of 73 total CTA+ samples evaluable for F1CDx testing, 68 were F1CDx+ and 5 were F1CDx− (Supplementary Table S4). The resulting PPA was 93.2% (Wilson score two-sided 95% CI, 85%–97%; Table 3). Of the 100 CTA− samples, 100 (100%) were also found to be *BRAF* V600E negative by the F1CDx test. The resulting NPA was 100% (Wilson score two-sided 95% CI, 96.3%–100%).

### Clinical validity of the F1LCDx test

The clinical efficacy of encorafenib and binimetinib combination therapy in patients selected by F1LCDx was based on the ORR by IRR (primary efficacy endpoint) in the PHAROS trial. For the treatment-naïve cohort, the ORR in the F1LCDx+/CTA+ population [74.2% (95% CI, 56.8%–86.3%)] was similar to that in the CTA+ population [74.6% (95% CI, 62.2%–83.9%); Table 4]. For the previously treated cohort, the ORR in the F1LCDx+/CTA+ population [35.3% (95% CI, 17.3%–58.7%)] was numerically lower than that in the CTA+ population [46.2% (95% CI, 31.6%–61.4%)]; however, the 95% CIs for these data overlap, and the numerical difference may be attributed to the smaller sample size as a result of F1LCDx-unevaluable samples. When the treatment-naïve and previously treated cohorts were combined, the ORR in the F1LCDx+/CTA+ population [60.4% (95% CI, 46.3%–73%)] was similar to that of the CTA+ population [63.3% (95% CI, 53.4%–72.1%)]. The ORR in the F1LCDx−/CTA+ population [57.6% (95% CI, 40.8%–72.8%)] was numerically lower than that in the CTA+ population. When the F1LCDx−/CTA+ population was stratified by TF, the group with a TF < 1% had a numerically higher ORR [70.4% (95% CI, 51.5%–84.1%)] compared with the group with a TF ≥ 1% (ORR of 0%), although the sample size of this group was very small (additional details in “Discussion”). Fourteen patients with CR or PR were unevaluable for F1LCDx testing because of sample unavailability or DNA extraction failure and were included in the F1LCDx-unevaluable/CTA+ population, which had an ORR of 82.4% (95% CI, 59%–93.8%).

**Table 4.** Primary efficacy in the F1LCDx test subpopulations.

​Treatment history	CTA+	F1LCDx+/CTA+	F1LCDx−/CTA+	F1LCDx−/CTA+ (TF < 1%)	F1LCDx−/CTA+ (TF ≥ 1%)	F1LCDx unevaluable/CTA+	F1LCDx+
Treatment naïve							
Number of patients	59	31	19	16	3	9	NA
Number of events (CR or PR)	44	23	13	13	0	8	NA
ORR, % (two-sided 95% CI)	74.6 (62.2–83.9)	74.2 (56.8–86.3)	68.4 (46–84.6)	81.2 (57–93.4)	0 (– to –)^a^	88.9 (– to –)^a^	74.2 (58.8–89.6)^b^
Previously treated							
Number of patients	39	17	14	11	3	8	NA
Number of events (CR or PR)	18	6	6	6	0	6	NA
ORR, % (two-sided 95% CI)	46.2 (31.6–61.4)	35.3 (17.3–58.7)	42.9 (21.4–67.4)	54.5 (28–78.7)	0 (– to –)^a^	75 (– to –)^a^	35.3 (12.6–58)^b^
Treatment naïve + previously treated							
Number of patients	98	48	33	27	6	17	NA
Number of events (CR or PR)	62	29	19	19	0	14	NA
ORR, % (two-sided 95% CI)	63.3 (53.4–72.1)	60.4 (46.3–73)	57.6 (40.8–72.8)	70.4 (51.5–84.1)	0 (– to –)^a^	82.4 (59–93.8)	60.4 (46.6–74.3)^b^

Abbreviation: NA, not available.

a CI was not calculated as the sample size is <10.

b CI was calculated based on the variance of the estimated F1LCDx+ efficacy.

### Clinical validity of the F1CDx test

The clinical efficacy of encorafenib and binimetinib combination therapy in patients selected by F1CDx was based on the ORR by IRR in the PHAROS trial. In this study, the ORR was calculated from 92 CTA+ samples and each subpopulation according to F1CDx *BRAF* V600 status (Supplementary Table S4). For the treatment-naïve cohort, the ORR in the F1CDx+/CTA+ population [82.9% (95% CI, 68.7%–91.5%)] was numerically higher than that in the CTA+ population [75.4% (95% CI, 62.9%–84.8%); Supplementary Table S5]. For the previously treated cohort, the ORR in the F1CDx+/CTA+ population [51.9% (95% CI, 34%–69.3%)] was numerically higher than that in the CTA+ population [45.7% (95% CI, 30.5%–61.8%)]; however, the 95% CIs for these data overlap. When the treatment-naïve and previously treated cohorts were combined, the ORR in the F1CDx+/CTA+ population [70.6% (95% CI, 58.9%–80.1%)] was numerically higher than that in the CTA+ cohort [64.1% (95% CI, 54%–73.2%)]; however, the data have overlapping 95% CIs. Nine patients with CR or PR were unevaluable for F1CDx testing (F1CDx unevaluable/CTA+ population) and had an ORR of 47.4% (95% CI, 27.3%–68.3%).

### Sensitivity analyses

#### F1LCDx sensitivity analysis

After including imputed data, the estimated median PPA between CTAs and F1LCDx was 60.8%, and the median PPVs for the 2%, 4%, and 8% prevalence parameters were all 100% (Supplementary Table S6). Using imputed complete data for the F1LCDx+/CTA+ population, the median ORRs in treatment-naïve and previously treated patients were 76.3% and 43.5%, respectively. When the cohorts were combined, the median ORR was 64.4% (Supplementary Table S7). The estimated drug efficacy in the F1LCDx+/CTA+ population in the combined cohort [ORR, 64.2% (95% CI, 51.8%–76.6%)] and individual cohorts [ORR in treatment naïve: 76.3% (95% CI, 62.4%–90.3%); ORR in previously treated: 43.3% (95% CI, 22.3%–64.3%)] was comparable with that in the F1LCDx+/CTA+ population (Supplementary Table S8).

#### F1CDx sensitivity analysis

After including imputed data, the estimated median PPA between CTAs and F1CDx was 92.5% (Supplementary Table S9). All results were the same across the 4 different prevalence values. After imputing the F1CDx status for the F1CDx-unevaluable samples, the median ORRs in the F1CDx+/CTA+ population in the treatment-naïve, previously treated, and combined treatment-naïve and previously treated cohorts were 82%, 45.7%, and 67.1%, respectively (Supplementary Table S10). The estimated drug efficacy in the F1CDx+ population based on observed and imputed data for a given sensitivity value is shown in Supplementary Table S11.

## Discussion

The F1LCDx and F1CDx companion diagnostic tests were simultaneously approved by the FDA to identify patients with *BRAF* V600E–mutant metastatic NSCLC who may benefit from the combination treatment of encorafenib with binimetinib (13). Using either a blood- or a tissue-based test to determine therapy candidacy expands access to this combination treatment option and provides flexibility for both patients and physicians, especially in cases in which tumor tissue is not readily available for testing and/or the diagnostic results from blood or tissue samples cannot be provided in a timely manner. These findings support a complementary testing approach rather than a replacement strategy. Plasma-based testing may provide a rapid and minimally invasive option to identify patients with *BRAF* V600E–mutant metastatic NSCLC, whereas tissue-based testing remains important in cases of negative, unevaluable, or low-shedding plasma results and may provide additional clinicopathologic context.

The results of this clinical bridging study showed the concordance between CTAs and F1LCDx samples with sufficient ctDNA shed [defined as a TF ≥ 1%; PPA of 83.3% (Wilson score two-sided 95% CI, 68.1%–92.1%); and NPA of 100% (Wilson score two-sided 95% CI, 91.8%–100%)] was consistent with higher TF samples performing better in the F1LCDx assay (30, 31). These results align with previous studies investigating the sensitivity of F1LCDx to detect short variant or rearrangement driver alterations identified in a tissue biopsy by F1CDx from the same patient (the PPA ranged from 49% to 86%). Furthermore, PPA values were consistently at or near 100% in cases in which the TF was ≥1% or ≥10%, respectively (30, 31). It should be noted that sensitivity varied with ctDNA shed, which can vary by cancer type (31, 32), and may have affected the results reported in our study. In this study, TF was primarily informative for plasma assay sensitivity, rather than as a discriminator of *BRAF* V600E prevalence. This is because the PHAROS clinical trial cohort was selected as *BRAF* V600E positive by CTA. Consistent with TF being informative for plasma assay sensitivity in this study, the PPA of F1LCDx was higher in samples with TF ≥ 1% than in those with TF < 1%. There was high concordance [PPA of 93.2% (Wilson score two-sided 95% CI, 85%–97%) and NPA of 100% (Wilson score two-sided 95% CI, 96.3%–100%)] between the CTAs and the F1CDx in detecting *BRAF* V600E in samples prospectively collected from patients in the phase II PHAROS trial. Of note, 4 of the 5 samples that were both F1CDx− and CTA+ would have received qualified F1CDx clinical reports because of sample quality issues, indicating that sensitivity for variant detection, including short variants, was potentially reduced. In the clinical setting, qualified reports include instructions for repeat testing. When these samples are excluded from the current analysis, the PPA increased to 98.6% (Wilson score two-sided 95% CI, 92.2%–99.7%). Given the high likelihood that F1CDx could detect alterations in the approximately 40% of samples that were negative by F1LCDx, and in line with FDA recommendations confirmatory tissue biopsy testing may be warranted if F1LCDx results are negative (33) and as seen in this study, particularly for samples with TF < 1%. Taken together, these findings support the complementary use of plasma- and tissue-based testing in clinical practice.

The clinical validity of F1LCDx was shown by estimating the ORR in the F1LCDx+/CTA+ population, which was similar to that observed in the CTA+ population for the combined cohorts. Although PFS is a clinically relevant endpoint, the present retrospective clinical bridging analysis was designed to evaluate clinical validity primarily by ORR by IRR. Furthermore, this was a single-arm study with no comparison group and small sample sizes, which would limit the interpretability of subgroup PFS analyses. Additional subgroup analyses of PFS according to F1LCDx and F1CDx status may be informative, but these analyses were not prespecified for the current study. In addition, the ORR in the F1LCDx+/CTA+ population increased following the sensitivity analysis and was comparable with that in the CTA+ population, showing the robustness of the F1LCDx assay after imputing data for the missing F1LCDx status. ORR results in the treatment-naïve cohort showed similar comparability between the F1LCDx+/CTA+ and CTA+ populations. The ORR for the F1LCDx+/CTA+ population was numerically lower in patients with previous treatment than the ORR in the CTA+ population; however, the F1LCDx+/CTA+ ORR improved after data imputation. The 95% CIs for both datasets overlapped, so the noted numerical discordance should be interpreted with caution as it may be due to small patient numbers in the subgroup or the missing contribution to the ORR from the 6 responders in the F1LCDx-unevaluable group that had an inherently higher ORR. The relatively favorable outcomes in patients with TF < 1% may reflect lower ctDNA burden, which may be associated with more favorable disease biology; consistent with this, a retrospective study reported that ctDNA detection and levels were generally higher in late-stage NSCLC than in early-stage NSCLC (34). Given the small sample sizes of the subgroups in the present study, these findings should be considered descriptive and interpreted cautiously. The imputed drug efficacy in the combined treatment naïve and previous treatment F1LCDx+ population in the sensitivity analysis was similar to the combined cohort F1LCDx+/CTA+ estimate from the ORR analysis and was comparable with the CTA+ efficacy. In addition, we further explored the F1LCDx−/CTA+ group by stratifying by a TF < 1% and a TF ≥ 1%. We observed that 6 patients with TF ≥ 1% had no objective response to the encorafenib in combination with binimetinib, suggesting that the CTA positivity could have been false positives in these cases, although the sample size was too small to make any definitive conclusions. On the other hand, patients with a TF < 1% had an ORR of 70.4%, whereas those with a TF ≥ 1% had a response, suggesting low ctDNA shed in those patients and subsequently poor detection by F1LCDx. Patients with TF < 1% and negative driver alterations should undergo follow-up tissue testing using an assay such as F1CDx. This emphasizes the value of a biomarker such as TF to guide testing decisions for patients. Together, these findings support the clinical utility of F1LCDx in identifying patients with *BRAF* V600E–mutant NSCLC who may benefit from encorafenib and binimetinib combination therapy, given the results for other oncogenic drivers and their findings, particularly the ability to identify point mutations versus rearrangements.

The clinical validity of F1CDx was shown by estimating the ORR in the F1CDx+/CTA+ population, which was numerically higher than that observed in the CTA+ population. Again, overlapping 95% CIs and the small sample size may affect the interpretation of these data. In addition, the imputed drug efficacy in the combined treatment naïve and previously treated F1CDx+ population in the sensitivity analysis was close to the combined cohort F1CDx+/CTA+ estimate from the ORR analysis and was comparable with the CTA+ efficacy. As with the F1LCDx assay, the sensitivity analysis demonstrated the robustness of the F1CDx assay after imputing data for the missing F1CDx status. Interestingly, in 3 of the 5 patients who were F1CDx−/CTA+, a *KRAS* driver alteration was also detected by F1CDx. Of these 3 *KRAS-*positive cases, 2 patients had no objective response to encorafenib in combination with binimetinib, whereas 1 patient had a PR, suggesting that the 2 cases with no response may have been false positives. Detailed information on the local CTA methodology and *BRAF* allele frequency was not uniformly available for these discordant cases and therefore could not be systematically evaluated in the present study.

Patients who are not able to provide evaluable tumor tissue for genomic testing or who face significant health risks from the procedures required to obtain a tumor biopsy may have limited options; a well-validated and reliable liquid biopsy test could provide a pragmatic alternative to tissue biopsy for identifying patients with metastatic NSCLC who might benefit from encorafenib in combination with binimetinib. The use of a multigene panel such as F1LCDx enables identification of other alterations in metastatic NSCLC [e.g., epidermal growth factor receptor (*EGFR*), KRAS proto-oncogene, GTPase (*KRAS*), and anaplastic lymphoma kinase (*ALK*)] or across NSCLC subtypes [e.g., NK1 homeobox 2 (*NKX1-2*)] that are relevant to diagnosis and selection of potential treatment options for patients (35–38). Concomitant biomarkers may provide additional biological context for the interpretation of molecular results. Co-occurring oncogenic alterations may help explain discordant results or lack of response to targeted therapy. In addition, immunotherapy-related biomarkers, such as PD-L1 expression and tumor mutational burden, were not evaluated as part of the study protocol, and their association with clinical outcomes could not be assessed. In addition to supporting the identification of patients with *BRAF* V600E mutations who may benefit from treatment with encorafenib in combination with binimetinib, a comprehensive genomic profiling test such as F1LCDx may provide an opportunity to monitor mutational signatures associated with acquired resistance alterations (39, 40). An additional potential advantage of plasma-based testing is the ability to perform serial assessments over time. In principle, dynamic evaluation of *BRAF* V600E in ctDNA may help monitor molecular response, residual disease burden, or emergence of resistance mechanisms. However, longitudinal sampling was outside the scope of the present retrospective clinical bridging study.

In this retrospective clinical bridging study, the main diagnostic was a CTA, whereas the F1LCDx and F1CDx—NGS-based comprehensive genomic profiling assays—were performed on ctDNA extracted from blood and DNA from tumor tissue, respectively. This retrospective bridging study was therefore only possible in a subset of participants who had available plasma and tumor tissue samples.

Overall, this clinical bridging study supports the complementarity of the F1LCDx and F1CDx tests in identifying patients with *BRAF* V600E–mutant metastatic NSCLC. Furthermore, both are clinically valid assays for predicting an objective response in patients with *BRAF* V600E–mutant metastatic NSCLC who may be eligible for treatment with encorafenib in combination with binimetinib.

## Supplementary Material

Supplementary Table S1 Table S1. Demographics and clinical characteristics in the F1CDx-evaluable and -unevaluable populations

Supplementary Table S2 Table S2. Plasma sample sizes and sources for F1LCDx testing

Supplementary Table S3 Table S3. Concordance analysis results between CTA and F1LCDx tests

Supplementary Table S4 Table S4. Contingency table comparing BRAF V600E status between the CTA and F1CDx

Supplementary Table S5 Table S5. Primary efficacy in the F1CDx test subpopulations

Supplementary Table S6 Table S6. Summary statistics of F1LCDx test PPA and PPV after including imputed data

Supplementary Table S7 Table S7. Summary statistics of ORR for the F1LCDx+/CTA+ population (ƍ1) on imputed complete data

Supplementary Table S8 Table S8. Estimated drug efficacy for the F1LCDx+/CTA+ population (ƍ1) on imputed complete data

Supplementary Table S9 Table S9. Summary statistics of F1CDx test PPA and PPV after including imputed data

Supplementary Table S10 Table S10. Summary statistics of ORR for the F1CDx+/CTA+ population (ƍ1) on imputed complete data

Supplementary Table S11 Table S11. Estimated drug efficacy for the F1CDx+ population (ƍCDx+) on imputed complete data

Supplementary Methods PHAROS study design and participants, clinical validation, and F1CDx analyses

## Data Availability

Upon request, and subject to review, Pfizer will provide the data that support the findings of this study. Subject to certain criteria, conditions, and exceptions, Pfizer may also provide access to the related individual deidentified participant data. See https://www.pfizer.com/science/clinical-trials/trial-data-and-results for more information.
