# Supplementary material for: Clinical Validation of Tissue and Liquid Companion Diagnostics for BRAF V600E Detection in Non–Small Cell Lung Cancers from the PHAROS Study
Source: Cancer Res Commun. 2026 Jul 29;6(7):1814–24. doi: 10.1158/2767-9764.CRC-26-0102 (PMC13416939; doi:10.1158/2767-9764.CRC-26-0102)
Supplement: Supplementary Table S1 — Table S1. Demographics and clinical characteristics in the F1CDx-evaluable and -unevaluable populations [file crc-26-0102_supplementary_table_s1_suppst1.pdf]

Supplementary Table S1. Demographics and clinical characteristics in the F1CDx-evaluable and -unevaluable populations

	Treatment naive		Previously Treated	
**Covariate**	F1CDx-evaluable/CTA+ (n=46)	F1CDx-unevaluable/CTA+ (n=11)	F1CDx-evaluable/CTA+ (n=27)	F1CDx-unevaluable/CTA+ (n=8)
**Age**				
**Median (range), years**	66.5 (47-83)	70.0 (60-80)	68.8 (53-86)	71.5 (56-84)
**Sex, n (%)**				
**Female**	26 (56.5)	6 (54.6)	14 (51.9)	3 (37.5)
**Male**	20 (43.5)	5 (45.5)	13 (48.2)	5 (62.5)
**Race, n (%)**				
**American Indian/Alsakan Native**	1 (2.2)	0	0	0
**Asian**	2 (4.4)	1 (9.1)	4 (14.8)	0
**Black/African American**	0	1 (9.1)	1 (3.7)	1 (12.5)
**White**	42 (91.3)	9 (81.8)	22 (81.5)	7 (87.5)
**Unknown**	1 (2.2)	0	0	0
**ECOG PS, n (%)**				
**0**	16 (34.8)	1 (9.1)	4 (14.8)	2 (25.0)
**1**	30 (65.2)	10 (90.9)	23 (85.2)	6 (75.0)
**Smoking status, n (%)**				
**Current smoker**	6 (13.0)	2 (18.2)	4 (14.8)	0
**Former smoker**	27 (58.7)	6 (54.6)	16 (59.3)	5 (62.5)
**Never smoked**	13 (28.3)	3 (27.3)	7 (25.9)	3 (37.5)
**Tissue sample site, n (%)**				
**Metastatic**	14 (43.8)	6 (60.0)	9 (45.0)	3 (50.0)
**Primary**	18 (56.3)	4 (40.0)	11 (55.0)	3 (50.0)
**Not available**	0	0	0	0
**Tissue handling, n (%)**				
**Core needle/excisional biopsy**	22 (48.9)	8 (80.0)	19 (70.4)	5 (62.5)
**Fine needle aspiration**	12 (26.7)	0	5 (18.5)	1 (12.5)
**Resection**	9 (20.0)	1 (10.0)	2 (7.4)	0
**Other**	2 (4.4)	1 (10.0)	1 (3.7)	2 (25.0)
**Not available**	1 (2.2)	0	0	0

CTA, clinical trial assay; ECOG PS, Eastern Cooperative Oncology Group performance status; F1CDx, FoundationOne® CDx.
