# Supplementary material for: Clinical Validation of Tissue and Liquid Companion Diagnostics for BRAF V600E Detection in Non–Small Cell Lung Cancers from the PHAROS Study
Source: Cancer Res Commun. 2026 Jul 29;6(7):1814–24. doi: 10.1158/2767-9764.CRC-26-0102 (PMC13416939; doi:10.1158/2767-9764.CRC-26-0102)
Supplement: Supplementary Table S2 — Table S2. Plasma sample sizes and sources for F1LCDx testing [file crc-26-0102_supplementary_table_s2_suppst2.pdf]

**Supplementary Table S2. Plasma sample sizes and sources for F1LCDx testing**

| Plasma sample sources | Total no. of patients | No. of failed or unavailable samples for F1LCDx testing | Total no. of F1LCDx-evaluable samples |
|---|---|---|---|
| **CTA+[a]** | 98 | 17[b] | 81 |
| **Procured CTA− samples tested by Cobas PCR assay** | 28 | 9[c] | 19 |
| **Procured CTA− samples tested by UW OncoPlex assay** | 39 | 9[c] | 30 |
| **CTA− from an FMI clinical archive** | 50 | 0 | 50 |
| **Total, n (%)** | 215 (100) | 35 (16.3) | 180 (83.7) |

CTA, clinical trial assay; F1LCDx, FoundationOne® Liquid CDx; FMI, Foundation Medicine, Inc.; PCR, polymerase chain reaction; UW, University of Washington.

[a]Six patients from the clinical trial were enrolled by F1CDx and were treated as CTA+.

[b]This number represents the 15 process failures plus 2 patients that were unevaluable due to lack of plasma for F1LCDx testing. See Figure 1.

[c]These are process failures for the procured plasma samples. See Figure 1.
