# Supplementary material for: Clinical Validation of Tissue and Liquid Companion Diagnostics for BRAF V600E Detection in Non–Small Cell Lung Cancers from the PHAROS Study
Source: Cancer Res Commun. 2026 Jul 29;6(7):1814–24. doi: 10.1158/2767-9764.CRC-26-0102 (PMC13416939; doi:10.1158/2767-9764.CRC-26-0102)
Supplement: Supplementary Table S3 — Table S3. Concordance analysis results between CTA and F1LCDx tests [file crc-26-0102_supplementary_table_s3_suppst3.pdf]

Supplementary Table S3. Concordance analysis results between CTA and F1LCDx tests

	Prevalence, %	Concordant result with CTA and F1LCDx test	Denominator[a]	Point estimate, % (two-sided 95% CI[b]), %
**PPA**	NA	48	81	59.3 (48.4, 69.3)
**NPA**	NA	99	99	100 (96.3, 100)
**Adjusted PPV**	2	NA	NA	100 (92.6, 100)
**Adjusted NPV**	2	NA	NA	99.2 (99.0, 99.4)
**Adjusted PPV**	4	NA	NA	100 (92.6, 100)
**Adjusted NPV**	4	NA	NA	98.3 (97.9, 98.7)
**Adjusted PPV**	8	NA	NA	100 (92.6, 100)
**Adjusted NPV**	8	NA	NA	96.6 (95.7, 97.4)

CI, confidence interval; CTA, clinical trial assay; F1LCDx, FoundationOne®Liquid CDx; NA, not available; NPA, negative percent agreement; NPV, negative predictive values; PPA, positive percent agreement; PPV, positive predictive values.

[a]The denominator for PPA is the total number of CTA+ samples among the F1LCDx-evaluable samples. The denominator for NPA is the total number of CTA− samples among the F1LCDx-evaluable samples.

[b]CI was calculated using the Wilson score method for PPA, NPA and PPV, while using the bootstrap method for the adjusted NPV.
