# Supplementary material for: Clinical Validation of Tissue and Liquid Companion Diagnostics for BRAF V600E Detection in Non–Small Cell Lung Cancers from the PHAROS Study
Source: Cancer Res Commun. 2026 Jul 29;6(7):1814–24. doi: 10.1158/2767-9764.CRC-26-0102 (PMC13416939; doi:10.1158/2767-9764.CRC-26-0102)
Supplement: Supplementary Table S4 — Table S4. Contingency table comparing BRAF V600E status between the CTA and F1CDx [file crc-26-0102_supplementary_table_s4_suppst4.pdf]

**Supplementary Table S4. Contingency table comparing *BRAF* V600E status between the CTA and F1CDx**

| | CTA+ | CTA− | Total |
|---|---|---|---|
| **F1CDx-evaluable** | 73 | 100 | 173 |
| **F1CDx+** | 68 | 0 | 68 |
| **F1CDx−** | 5[a] | 100 | 105 |
| **F1CDx-unevaluable** | 19[b] | 0 | 19 |
| **Total** | 92[c] | 100 | 192 |

BRAF, B-Raf proto-oncogene, serine/threonine kinase; CTA, clinical trial assay; F1CDx, FoundationOne®CDx.

[a]Four of the five samples that were F1CDx−/CTA+ would have had a qualified F1CDx test report due to sample quality issues. A qualified report indicates that sensitivity for variant detection, including short variants, is potentially reduced. In the clinical setting, this will be stated on the clinical report and repeat testing is recommended.

[b]This number represents the 10 samples with insufficient DNA mass, 5 samples that failed QC metrics, 3 samples that did not meet acceptance criteria, and 1 sample that had insufficient quantity remaining for F1CDx testing. See Figure 2.

[c]Six patients were enrolled by F1CDx and were not included in the concordance analysis.
