# Supplementary material for: Clinical Validation of Tissue and Liquid Companion Diagnostics for BRAF V600E Detection in Non–Small Cell Lung Cancers from the PHAROS Study
Source: Cancer Res Commun. 2026 Jul 29;6(7):1814–24. doi: 10.1158/2767-9764.CRC-26-0102 (PMC13416939; doi:10.1158/2767-9764.CRC-26-0102)
Supplement: Supplementary Table S5 — Table S5. Primary efficacy in the F1CDx test subpopulations [file crc-26-0102_supplementary_table_s5_suppst5.pdf]

Supplementary Table S5. Primary efficacy in the F1CDx test subpopulations

	CTA+	F1CDx+/ CTA+	F1CDx−/ CTA+	F1CDx-unevaluable/CTA+	F1CDx+[a]
**Treatment naive**					
**No. of patients**	57	41	5[c]	11	2
**No. of events (CR or PR)**	43	34	2	7	1
**ORR, % (two-sided 95% CI)**	75.4 (62.9, 84.8)	82.9 (68.7, 91.5)	40.0 (-, -)[b]	63.6 (35.4, 84.8)	50.0 (-, -)[b]
**Previously treated**					
**No. of patients**	35	27	0	8	4
**No. of events (CR or PR)**	16	14	0	2	2
**ORR, % (two-sided 95% CI)**	45.7 (30.5, 61.8)	51.9 (34.0, 69.3)	- (-, -)[c]	25.0 (-, -)[b]	50.0 (-, -)[b]
**Treatment naive + Previously treated**					
**No. of patients**	92	68	5*	19	6
**No. of events (CR or PR)**	59	48	2	9	3
**ORR, % (two-sided 95% CI)**	64.1 (54.0, 73.2)	70.6 (58.9, 80.1)	40.0 (-, -)[b]	47.4 (27.3, 68.3)	50.0 (-, -)[b]

CI, confidence interval; CR, complete response; CTA, clinical trial assay; F1CDx, FoundationOne®CDx; ORR, objective response rate; PR, partial response.

[a]F1CDx+ represents the F1CDx-enrolled sub-population.

[b]CI was not calculated since the sample size is <10.[c]ORR could not be estimated as there were zero samples in this group.

[c]Four of the five samples that were F1CDx−/CTA+ would have had a qualified F1CDx test report due to sample quality issues. A qualified report indicates that sensitivity for variant detection, including short variants, is potentially reduced. In the clinical setting, this will be stated on the clinical report and repeat testing is recommended.
