# Supplementary material for: Clinical Validation of Tissue and Liquid Companion Diagnostics for BRAF V600E Detection in Non–Small Cell Lung Cancers from the PHAROS Study
Source: Cancer Res Commun. 2026 Jul 29;6(7):1814–24. doi: 10.1158/2767-9764.CRC-26-0102 (PMC13416939; doi:10.1158/2767-9764.CRC-26-0102)
Supplement: Supplementary Table S6 — Table S6. Summary statistics of F1LCDx test PPA and PPV after including imputed data [file crc-26-0102_supplementary_table_s6_suppst6.pdf]

Supplementary Table S6. Summary statistics of F1LCDx test PPA and PPV after including imputed data

	PPA, %	PPV, %	PPV, %	PPV, %
**Prevalence parameter**	NA	2%	4%	8%
**Mean (min, max)**	61.2 (56.7, 65.0)	100 (100, 100)	100 (100, 100)	100 (100, 100)
**2.5%**	57.7	100	100	100
**Q1**	59.8	100	100	100
**Median**	60.8	100	100	100
**Q3**	62.9	100	100	100
**97.5%**	63.9	100	100	100

F1LCDx, FoundationOne®Liquid CDx; max, maximum; min, minimum; NA, not available; PPA, positive percent agreement; PPV, positive predictive values; Q, quartile.
