# Supplementary material for: Clinical Validation of Tissue and Liquid Companion Diagnostics for BRAF V600E Detection in Non–Small Cell Lung Cancers from the PHAROS Study
Source: Cancer Res Commun. 2026 Jul 29;6(7):1814–24. doi: 10.1158/2767-9764.CRC-26-0102 (PMC13416939; doi:10.1158/2767-9764.CRC-26-0102)
Supplement: Supplementary Table S7 — Table S7. Summary statistics of ORR for the F1LCDx+/CTA+ population (ƍ1) on imputed complete data [file crc-26-0102_supplementary_table_s7_suppst7.pdf]

Supplementary Table S7. Summary statistics of ORR for the F1LCDx+/CTA+ population ($\delta 1$) on imputed complete data

	Treatment naive	Previously treated	Treatment-naive + Previously treated
**Mean (min, max), %**	76.3 (74.3, 79.0)	43.3 (35.0, 48.0)	64.2 (60.7, 67.2)
**2.5%**	75.0	38.1	62.1
**Q1**	75.7	40.9	63.3
**Median, %**	76.3	43.5	64.4
**Q3**	77.1	45.8	65.0
**97.5%**	78.4	48.0	66.1

CTA, clinical trial assay; F1LCDx, FoundationOne®Liquid CDx; max, maximum; min, minimum; ORR, objective response rate; Q, quartile.
