# Supplementary material for: Clinical Validation of Tissue and Liquid Companion Diagnostics for BRAF V600E Detection in Non–Small Cell Lung Cancers from the PHAROS Study
Source: Cancer Res Commun. 2026 Jul 29;6(7):1814–24. doi: 10.1158/2767-9764.CRC-26-0102 (PMC13416939; doi:10.1158/2767-9764.CRC-26-0102)
Supplement: Supplementary Table S8 — Table S8. Estimated drug efficacy for the F1LCDx+/CTA+ population (ƍ1) on imputed complete data [file crc-26-0102_supplementary_table_s8_suppst8.pdf]

Supplementary Table S8. Estimated drug efficacy for the F1LCDx+/CTA+ population ($\delta 1$) on imputed complete data

	Treatment naive	Previously treated	Treatment-naive + Previously treated
**Median ORR, %**	76.3	43.3	64.2
**Two-sided 95% CI**	(62.4, 90.3)	(22.3, 64.3)	(51.8, 76.6)

CI, confidence interval; CTA, clinical trial assay; F1LCDx, FoundationOne®Liquid CDx; ORR, objective response rate.
