# Supplementary material for: Clinical Validation of Tissue and Liquid Companion Diagnostics for BRAF V600E Detection in Non–Small Cell Lung Cancers from the PHAROS Study
Source: Cancer Res Commun. 2026 Jul 29;6(7):1814–24. doi: 10.1158/2767-9764.CRC-26-0102 (PMC13416939; doi:10.1158/2767-9764.CRC-26-0102)
Supplement: Supplementary Table S9 — Table S9. Summary statistics of F1CDx test PPA and PPV after including imputed data [file crc-26-0102_supplementary_table_s9_suppst9.pdf]

Supplementary Table S9. Summary statistics of F1CDx test PPA and PPV after including imputed data

	PPA, %	PPV, %[a]
**Mean (min, max)**	92.5 (91.3, 93.5)	100
**2.5%**	91.3	100
**Q1**	92.4	100
**Median**	92.4	100
**Q3**	92.4	100
**97.5%**	93.5	100

F1CDx, FoundationOne®CDx; max, maximum; min, minimum; PPA, positive percent agreement; PPV, positive predictive values; Q, quartile.

[a]All PPV results were the same across all different prevalence values.
