# Supplementary material for: Clinical Validation of Tissue and Liquid Companion Diagnostics for BRAF V600E Detection in Non–Small Cell Lung Cancers from the PHAROS Study
Source: Cancer Res Commun. 2026 Jul 29;6(7):1814–24. doi: 10.1158/2767-9764.CRC-26-0102 (PMC13416939; doi:10.1158/2767-9764.CRC-26-0102)
Supplement: Supplementary Table S10 — Table S10. Summary statistics of ORR for the F1CDx+/CTA+ population (ƍ1) on imputed complete data [file crc-26-0102_supplementary_table_s10_suppst10.pdf]

Supplementary Table S10. Summary statistics of ORR for the F1CDx+/CTA+ population ($\delta 1$) on imputed complete data

	Treatment naive	Previously treated	Treatment-naive + Previously treated
**Mean (min, max), %**	81.7 (78.9, 83.7)	46.2 (43.8, 50.0)	67.0 (66.3, 67.9)
**2.5%**	80.4	44.1	66.3
**Q1**	82.0	45.7	67.1
**Median, %**	82.0	45.7	67.1
**Q3**	82.0	47.1	67.1
**97.5%**	83.7	48.9	67.9

CTA, clinical trial assay; F1CDx, FoundationOne®CDx; max, maximum; min, minimum; ORR, objective response rate; Q, quartile.
