# Supplementary material for: Clinical Validation of Tissue and Liquid Companion Diagnostics for BRAF V600E Detection in Non–Small Cell Lung Cancers from the PHAROS Study
Source: Cancer Res Commun. 2026 Jul 29;6(7):1814–24. doi: 10.1158/2767-9764.CRC-26-0102 (PMC13416939; doi:10.1158/2767-9764.CRC-26-0102)
Supplement: Supplementary Table S11 — Table S11. Estimated drug efficacy for the F1CDx+ population (ƍCDx+) on imputed complete data [file crc-26-0102_supplementary_table_s11_suppst11.pdf]

Supplementary Table S11. Estimated drug efficacy for the F1CDx+ population (δCDx+) on imputed complete data

c (%)[a]	Treatment naive ORR, % (95% CI)	Previously treated ORR, % (95% CI)	Treatment-naive + Previously treated ORR, % (95% CI)
**0**	79.8 (67.7, 92.0)	49.9 (32.4, 67.3)	67.7 (56.9, 78.4)
**30**	79.9 (67.8, 92.0)	50.6 (33.0, 68.2)	68.1 (57.4, 78.7)
**50**	80.2 (68.1, 92.3)	51.0 (33.5, 68.6)	68.0 (57.3, 78.7)
**70**	80.6 (68.7, 92.6)	51.5 (33.9, 69.1)	68.3 (57.6, 78.9)
**100**	81.4 (69.8, 93.0)	51.3 (33.7, 68.9)	68.9 (58.3, 79.4)

CI, confidence interval; CTA, clinical trial assay; F1CDx, FoundationOne®CDx; ORR, objective response rate.

[a]*c* is a sensitivity value for evaluating the clinical efficacy of F1CDx+/CTA− samples.
