# Supplementary material for: Clinical Validation of Tissue and Liquid Companion Diagnostics for BRAF V600E Detection in Non–Small Cell Lung Cancers from the PHAROS Study
Source: Cancer Res Commun. 2026 Jul 29;6(7):1814–24. doi: 10.1158/2767-9764.CRC-26-0102 (PMC13416939; doi:10.1158/2767-9764.CRC-26-0102)
Supplement: Supplementary Methods — PHAROS study design and participants, clinical validation, and F1CDx analyses [file crc-26-0102_supplementary_methods_suppsm.pdf]

**Clinical Validations of Tissue and Liquid Companion Diagnostics for Detection of *BRAF* V600E in Non-Small Cell Lung Cancer from the PHAROS Study**

## SUPPLEMENTARY METHODS

### *Overview of PHAROS study design and participants*

The PHAROS study design and primary analysis have been published previously [14]. Briefly, PHAROS (ClinicalTrials.gov: NCT03915951) is an ongoing, single-arm, open-label, multicenter, non-randomized phase 2 trial that enrolled patients between June 4, 2019 and June 2, 2022. Detailed inclusion and exclusion criteria for the PHAROS trial have been described and a study protocol is available [14]. Briefly, the trial included adult patients (≥18 years) with histologically confirmed stage IV or recurrent NSCLC, measurable disease (per Response Evaluation criteria in Solid Tumors version 1.1 [ RECIST v1.1]), and an ECOG PS of ≤1. Patients were enrolled in two cohorts: treatment naive and previously treated. The previously treated cohort consisted of patients with no more than 1 prior line of treatment, either a prior first-line platinum-based chemotherapy or platinum-based chemotherapy plus an anti–PD-1/PD-L1 inhibitor treatment. Patients with driver mutations other than BRAF V600 class I mutations, prior treatment with a BRAF or MEK inhibitor, untreated symptomatic brain metastasis, or leptomeningeal disease were excluded. Eligible patients (59 treatment-naive patients and 39 previously treated patients) were treated with oral encorafenib (450 mg daily) and oral binimetinib (45 mg twice daily) in 28-day cycles.

### *Clinical validation*

The primary endpoint was confirmed objective response rate (ORR), assessed according to RECIST v1.1 by independent radiology review (IRR) [14]. ORR is

defined as the proportion of patients with objective response of either confirmed complete response or partial response based on RECIST v1.1. Secondary endpoints included confirmed ORR by investigator assessment; duration of response, disease control rate, progression-free survival, and time to response by IRR and investigatory assessment; and overall survival and safety [14].

***Clinical bridging study design and samples for F1CDx analyses***

FoundationOne®CDx (F1CDx) is an FDA-approved next-generation sequencing-based companion diagnostic test that targets 324 cancer-related genes and can be used to identify patients who may benefit from treatment with approved therapeutic products [6].

F1CDx testing was performed on *BRAF* V600E–positive tumor tissue samples from the PHAROS trial from patients who were not enrolled by F1CDx, who had provided patient consent or the appropriate waiver, and who had sufficient formalin-fixed paraffin-embedded tissue material for testing [14]. Samples were required to meet sample inclusion criteria listed in the clinical trial protocol and to meet process-related acceptance criteria to be included in the analysis [14].

A total of 198 samples including clinical trial samples and Foundation Medicine, Inc (FMI) banked samples were included in this study. Of the 198 samples, 98 biomarker (*BRAF* V600E)–positive samples were from the PHAROS trial and 100 *BRAF* V600E-negative samples were randomly selected from a Foundation Medicine, Inc. clinical archive. Of the 98 patients who were BRAF V600E–positive, 59 were treatment-naive (1L) patients, 39 had received prior treatment (2L), and 6 were enrolled with the F1CDx assay. Those patients who were enrolled with the F1CDx assay were excluded from the concordance analysis as per the study protocol. Of the remaining 92 patients, 3 patient samples did not meet the minimum sample

acceptance criteria, and 1 patient sample did not have sufficient material remaining for F1CDx testing. As such, a total of 88 CTA+ samples were included in this study for F1CDx testing.
